# Prospective Power Calculations for the Four Lab Study of A Multigenerational Reproductive/Developmental Toxicity Rodent Bioassay Using A Complex Mixture of Disinfection By-Products in the Low-Response Region

**DOI:** 10.3390/ijerph8104082

**Published:** 2011-10-24

**Authors:** Cheryl A. Dingus, Linda K. Teuschler, Glenn E. Rice, Jane Ellen Simmons, Michael G. Narotsky

**Affiliations:** 1 Statistics and Information Analysis, Battelle Memorial Institute, 505 King Ave., Columbus, OH 43201, USA; E-Mail: DingusC@battelle.org; 2 NCEA/ORD/U.S. EPA, 26 W. ML King Dr. (MS-190), Cincinnati, OH 45268, USA; E-Mail: Teuschler.linda@epa.gov; 3 NHEERL/ORD/U.S. EPA, 109 T.W. Alxander Dr., MD B243-01, Research Triangle Park, NC 27711, USA; E-Mails: Simmons.jane@epa.gov (J.E.S.); Narotsky.michael@epa.gov (M.G.N.)

**Keywords:** power calculations, experimental design, drinking water, disinfection by-products (DBP), chemical mixtures, low response, low dose, Four Lab Study

## Abstract

In complex mixture toxicology, there is growing emphasis on testing environmentally representative doses that improve the relevance of results for health risk assessment, but are typically much lower than those used in traditional toxicology studies. Traditional experimental designs with typical sample sizes may have insufficient statistical power to detect effects caused by environmentally relevant doses. Proper study design, with adequate statistical power, is critical to ensuring that experimental results are useful for environmental health risk assessment. Studies with environmentally realistic complex mixtures have practical constraints on sample concentration factor and sample volume as well as the number of animals that can be accommodated. This article describes methodology for calculation of statistical power for non-independent observations for a multigenerational rodent reproductive/developmental bioassay. The use of the methodology is illustrated using the U.S. EPA’s Four Lab study in which rodents were exposed to chlorinated water concentrates containing complex mixtures of drinking water disinfection by-products. Possible experimental designs included two single-block designs and a two-block design. Considering the possible study designs and constraints, a design of two blocks of 100 females with a 40:60 ratio of control:treated animals and a significance level of 0.05 yielded maximum prospective power (~90%) to detect pup weight decreases, while providing the most power to detect increased prenatal loss.

## 1. Introduction

Toxicological investigation of environmental chemical mixtures is evolving, with attention focused on defined mixtures, involving a limited number of chemicals, and complex environmental mixtures, involving a large number of chemicals and, typically, an unidentified fraction. Dosing regimens are being developed to evaluate the toxicity of complex mixtures using approaches consistent with human environmental exposures that are typically much lower than those used in traditional toxicology studies. Consequently, newer studies are designed to evaluate the toxicity of complex mixtures (1) with the inclusion of low, environmentally relevant dose levels; (2) with the relative proportions of component chemicals similar to those measured in environmental samples; and, (3) with approaches that maintain the chemically unidentified components of the mixture [1,2]. This type of study design differs from many traditional defined-mixture studies, conducted mostly on binary mixtures at relatively high dose levels where adverse effects are more readily detected if present, but whose relevance to human health risks associated with low environmental exposures is often unclear. This newer type of study design has been proposed specifically for application to drinking water disinfection by-products (DBPs) [3–5].

Disinfection of drinking water for microbial contamination provides an essential public health benefit in reduction of water-borne disease. However, oxidizing disinfectants react with materials in the source water resulting in the formation of a wide variety of DBPs. DBP mixtures are highly complex, containing numerous chemicals not routinely measured and many that are unknown; approximately 50% of the total organic halide compounds formed when water is disinfected remains unidentified [6–8]. Some epidemiologic studies report adverse developmental effects associated with exposure to DBPs, including low birth weight at term, small for gestational age [9,10], and stillbirths [11,12]. Epidemiologic evidence is mixed regarding associations of DBPs with spontaneous abortion [13,14]. Toxicity bioassays have been conducted on approximately 35 individual DBPs and a limited number of DBP mixtures [15–19]; some of the tested DBPs and DBP mixtures were shown to be reproductive or developmental toxicants in experimental animals.

Because concerns identified from epidemiologic studies on whole DBP mixtures cannot be readily addressed by investigating either individual DBPs or simple, defined DBP mixtures, scientists from four of the national laboratories and centers of the U.S. EPA’s Office of Research and Development have developed and, along with extramural partners, undertaken a research project (the Four Lab Study) that integrates toxicological and chemical evaluation of environmentally realistic complex mixtures of DBPs [4,5,20]. For complex DBP mixtures formed by chlorination, improved understanding of *in vivo* reproductive/developmental toxicology is a priority; consequently, the Four Lab study included a multigenerational reproductive/developmental toxicity rodent bioassay. Preparation for this experiment included: conducting a phased series of experiments involving water concentration, toxicology, and chemistry [8,21–25]; development of new risk assessment methodology [26]; and conducting developmental toxicity screens on sodium, sulfate, and concentrated DBPs [27].

The resulting database of toxicological and analytical chemistry data on the whole DBP mixture provides important information for health risk assessment of DBPs [26]. Risk assessment investigations include the analysis of associations between positive assay results (e.g., delays in attaining puberty) and dose level of specific DBPs (e.g., dichloroacetic acid) or groups of DBPs (e.g., trihalomethanes) identified within the whole mixture. Using existing dose-response data on individual chemicals, statistical models can then be used to test whether the observed toxicity of the whole mixture can be attributed to known DBPs, their joint toxic action, or to the unidentified fraction of the DBP mixture [26]. These distinctions are important for informed decisions with regard to controlling the levels of specific DBPs or groups of DBPs in finished drinking water, resulting in the production of clean, safe water. To allow for expanded use of the results, statistical and toxicological criteria have been developed to determine the “sufficient similarity” of DBP mixture composition and toxicity potential among finished drinking waters [28]. Using such criteria, it may be possible to extrapolate Four Lab Study results on the chlorinated concentrates to evaluate the potential for health effects from different DBP mixtures that could arise from: (1) treatment with other types of disinfectants (e.g., chloramination), (2) differences among source waters (e.g., differences in NOM) or (3) differences in treatment practices across treatment plants or over time within the same treatment plant.

Experimental constraints considered in the design of the multigenerational bioassay included: the number of dams that could be accommodated at one time (maximum of 100); the extent that water could be concentrated while retaining palatability and conserving organics (a concentration factor of 136× for total organic carbon was achieved for use in the multigenerational bioassay [29]); and, the quantity of concentrate that would be produced (~1,500 liters). Designing a meaningful study given these constraints required careful attention to statistical power.

In the multigenerational bioassay, timed-pregnant Sprague-Dawley rats comprising the parental (P_0_) generation would be assigned randomly to either a control group or a treatment group which would consume chlorinated water concentrate. Each P_0_ dam was to deliver a litter (F_1_ generation). An issue addressed in the present work was whether to breed one or two females from each F_1_ litter to a non-sibling F_1_ male from the same exposure group to produce the F_2_ generation. Priority study endpoints were prenatal loss (number of uterine implantation sites minus number of live pups at birth, divided by implantation sites) and pup birth weight. In comparison with epidemiologic endpoints of concern, prenatal loss is analogous to spontaneous abortion, whereas reduced pup weight is analogous to small for gestational age and term low birth weight.

U.S. EPA testing guidelines for reproductive toxicity call for 20 pregnant females per group as the standard protocol for single chemical bioassays [30]. The guidelines also state “the highest dose level should induce toxicity in the parental (P) animals and intermediate dose levels should produce minimal observable toxic effects. The lowest dose level should not produce any evidence of toxicity” [30]. Under these guidelines, the requirement for toxicity in the high and middle dose levels ensures adequate statistical power to detect effects, but not necessarily at the low dose. In contrast, in the multigenerational bioassay whose design was the goal of this paper, pregnant animals were to be exposed to much lower (*i.e.*, environmentally relevant) DBP concentrations than the guidelines suggest. Thus, study design, particularly estimation of statistical power, was essential to optimize sample sizes within the experimental constraints, so that effects, if present, would be detected, increasing the utility of the experimental results for risk assessment. This article describes the development and application of the methodology used for calculating statistical power for non-independent observations for this bioassay with chlorinated water concentrates in the Four Lab Study, taking into account the multigenerational bioassay design as well as constraints on sample size, concentration factor, and sample volume.

## 2. Methods

### 2.1. Statistical Methods

In this study, the individual pups within each litter represent repeated measurements that are not independent. A compound symmetric correlation structure was assumed, so that the correlation between any two pups in a litter was equal. Power and sample size calculations must account for the correlation; therefore, the methodology developed by Rochon [31] was followed.

As with less complicated sample size procedures, estimates of the group means and variances were required to calculate power. For the generalized-estimating equation (GEE)-adapted sample size methodology, estimates for the correlation between measurements and the over-dispersion factor (if appropriate) were also required. Estimates were derived by modeling data from Narotsky *et al.* [27]. Once these estimates were determined, a sequence of equations provided by Rochon [31] were applied that led to a calculation for either sample size or power. The covariance matrix was calculated for the set of repeated measurements and used with the gradient matrix to calculate the “model-based” covariance matrix for the weighted least squares estimator of the vector of treatment effect parameters. The final calculations were then based on the Wald test statistic, which is a function of the sample size, the effect size of interest, and the model-based covariance matrix. Under the alternative hypothesis, the Wald test statistic asymptotically follows a non-central chi-square distribution; the appropriate non-centrality parameter can be computed so that the test has a specified power and significance level desired for the final analysis and set equal to a function of the sample size, the effect size of interest, and the model-based covariance matrix. Because the Wald test statistic was being used, the power calculations were for testing the null hypothesis *H_0_:β_1_* *= β_2_*, *versus* the alternative hypothesis *H_α_:β_1 ≠_β_2_*, where *β_1_* and *β_2_* are the means of the endpoint within the control and treatment groups, respectively. The power calculations presented for pup weight are for testing the two-sided hypothesis, with the significance level α = 0.05. For prenatal loss, the *a priori* hypothesis is that treatment can only reduce the number of surviving progeny, thus, the power calculations presented for this endpoint are for testing the one-sided hypothesis, with the significance level of 2α = 0.10.

Pup weight at birth and prenatal loss were the focus of the power calculations for the Four Lab study. After conducting the study, statistical tests are to be performed independently for these two endpoints, and the Type I error rate will be set to α = 0.05 for each individual test. Based on the data of Narotsky *et al.* [27], the experimental design was optimized for detection of a 0.6 g difference in mean pup weight and a 7.1 percentage point difference in mean prenatal loss which is equivalent to a 1.9 treatment-to-control ratio in mean prenatal loss. The summary statistics for these endpoints, calculated from the raw data, are shown in Table 1. Modeled estimates based on these data were used to develop the power calculations; these differ only slightly from the summary statistics in Table 1.

## 3. Model

For analyzing pup weight, male and female pups were considered separately as well as combined; the results presented in this paper are for the combined male and female pups. The litter was treated as the experimental unit, with each pup within a litter representing a repeated measurement. A linear model was assumed for the data, so that:

(1) $$y_{ijk}=\beta_{i}x_{ij}+{ɛ}_{ijk}$$

where *y_ijk_* is the weight of the *k*^th^ live pup in the *j*^th^ litter of the *i*^th^ group (where *i =* 1 for the control group and *i =* 2 for the treatment group):

(2) $$x_{1j}=\{\begin{array}{ll} 1 & \text{if }i=1\\ 0 & \text{otherwise} \end{array}$$

and:

(3) $$x_{2j}=\{\begin{array}{ll} 1 & \text{if }i=2\\ 0 & \text{otherwise} \end{array}$$

In this model, the pup weights, *y_ijk_*, were assumed to follow a Gaussian distribution with mean *β_i_* and common variance-covariance matrix ∑ within litters. A constant correlation was assumed between the weights of all pups from the same litter (*i.e.*, a compound symmetric correlation structure), while weights of pups from different litters were assumed to be uncorrelated (independent).

An additional key endpoint, prenatal loss, was also examined with respect to power. For prenatal loss, litter again represented the experimental unit, with each implantation site representing a repeated measurement. The endpoint was then coded as:

$$Y_{ijk}=\{\begin{array}{l} 1\;\text{if the }k^{th}\;\text{implant site of the }j^{th}\;\text{dam in the }i^{th}\;\text{group results in a live birth}\\ 0\;\text{if the }k^{th}\;\text{implant site of the }j^{th}\;\text{dam in the }i^{th}\;\text{group does not result in a live birth} \end{array}$$

where *i* equals 1 (control) or 2 (treatment).

If *π_i_* denotes the mean prenatal loss probability among dams in the *i*^th^ group, then under a linear model:

(4) $$\pi_{i}=\beta_{i}x_{ij}$$

where *x*_1_*_j_* and *x*_2_*_j_* are defined as above. Alternatively, when a linear logistic model was assumed for the data, then:

(5) $$\text{log}\left(\frac{\pi_{i}}{1-\pi_{i}}\right)=\beta_{i}x_{ij}$$

where *x*_i_*_j_* and *x*_2_*_j_* are defined as above.

The linear and linear logistic models differ based upon the alternative hypotheses being tested. In both the linear and linear logistic models for prenatal loss a binomial error distribution was assumed for the model. In the linear model, the alternative hypothesis *H_α_:β_1_* ≠ *β_2_* represents the difference between the prenatal loss proportions of the control and treatment groups (e.g., the prenatal loss for the treatment group is 7.1 percentage points greater than the prenatal loss for the control group). In contrast, under the linear logistic model, the alternative hypothesis *H_α_*:*β_1_* ≠ *β_2_* represents the difference between the log odds of the control and treatment groups, which for small *π_i_* is approximately equal to the ratio of the prenatal loss proportions of the treatment and control groups (e.g., the prenatal loss for the treatment group is approximately twice the prenatal loss for the control group). Therefore, with respect to the biological interpretation of the endpoint, the specific question of interest, *i.e.*, an absolute increase over control *vs.* a proportional increase relative to control, is important to the appropriate model specification. The results for the two tests should be asymptotically equivalent under the null hypothesis, but sensitivity may differ under different alternative hypotheses. Results for both models are presented here.

For both the pup weight data and the prenatal loss data, an explicit relationship exists between the over-dispersion parameter, which represents the proportion of the observed variability that is due to the correlation among the observations, and the intra-litter correlation. For both distributions, the over-dispersion parameter, *ψ*, can be expressed as a function of the intra-litter correlation, *ρ*:

(6) $$\psi_{ij}=1+(n_{ij}-1)\rho$$

where *n_ij_* is the number of pups in the *j^th^* litter of the *i^th^* group. From this equation it follows that *ψ_ij_* = 1 if and only if *ρ* = 0 or *n_ij_* = 1. For each scenario examined, the intra-litter correlation *ρ* was specified based on the pilot data, and the dispersion parameter *ψ* was calculated according to the above equation. By doing this, the estimates for *ρ* given *ψ* closely matched those observed from modeling the Narotsky *et al.* [27] data.

### 3.1. Calculations and Assumptions

The number of P_0_ dams available for study was limited to 100 dams per block where experimental blocks were logistically constrained to being evaluated sequentially over time. Therefore, the problem was that of determining whether the study would have sufficient power to detect the treatment effects of interest (*i.e.*, a 0.6 g difference in mean pup weight and a 7.1 percentage point difference in mean prenatal loss or 1.9 treatment-to-control ratio in mean prenatal loss), with respect to the key endpoints of pup weight and prenatal loss. The targeted level of power was 80%, while maintaining a 0.05 significance level.

In each block, 100 dams would be divided into two groups: a control group and a treatment group receiving water concentrate containing a complex mixture of DBPs. In this study, one treatment group would be used due to the limited amount of water concentrate available.

The expected control group and treatment group means were estimated from the Narotsky *et al.* [27] data (Table 1); the variances and intra-litter correlations were also estimated from these pilot data. The modeled estimates used to develop power calculations differed only slightly from the statistics calculated from the raw data (Table 1). Litter size and the number of implantation sites were set equal to the average litter size and number of implantation sites based on the preliminary study data.

Having equal numbers of repeated measurements per experimental unit was a necessary assumption for the implementation of Rochon’s [31] methodology. Therefore, it was assumed that an equal number of live pups would be produced in each litter for all scenarios considered with respect to pup weight. Similarly, for prenatal loss, it was assumed that all females would produce an equal number of implantation sites. These assumptions are unlikely to be realized in practice, but were expected to have little impact on the observed power of the study.

### 3.2. Initial Designs

#### Single-Block Design, One F_1_ Female per Litter

**Figure 1:**
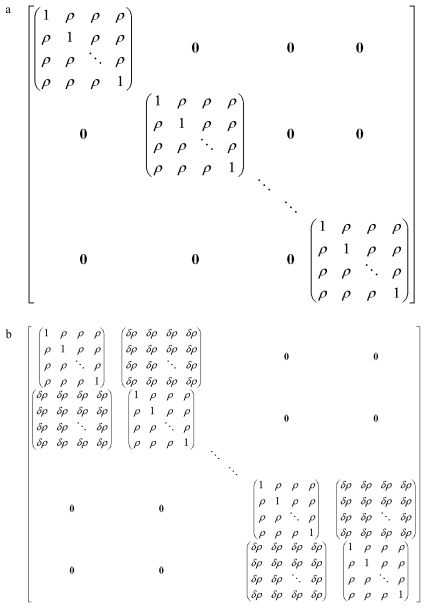
(**a**) Compound symmetric correlation structure assumed for design with one female per litter bred. The correlation between any two pups in a litter was assumed to be *ρ*. (**b**) Correlation structure assumed for design with two females per litter bred. The correlation between any two pups in a litter was assumed to be *ρ*, and the correlation between any two pups from litters of related females was assumed to be *δρ* where 0 < *δ*≤ 1.

The first design considered was a single-block design in which one F_1_ female rat per litter would be bred. In this design, a maximum of 100 dams would be available for assignment to the two groups (control and treatment). The compound symmetric correlation structure was used for pups within litters, and pups from different litters were assumed to be independent. The correlation matrix is given in Figure 1a. This design has the advantage of simplicity, both of execution and analysis.

The cohort size of 100 dams was sufficient to achieve the desired 80% power to detect a 0.6 g difference in average pup weight between the control and treatment groups at a significance level of 0.05 for all cases considered except one. With this simple design, greater than 99% power can be achieved with 50 dams assigned to each of the control and treatment groups, or with a 40:60 control:treatment group ratio assignment.

The single-block design failed to produce a sufficient level of power to detect a treatment effect on prenatal loss across the effect size, litter size, and correlation scenarios considered. Only when the intra-litter correlation was assumed to be zero (a very unlikely assumption) would 100 dams be sufficient to achieve the desired level of power. When the intra-litter correlation was assumed to be non-zero, the maximum achievable power, regardless of whether equal or unequal allocation of the dams occurred, was substantially lower than the desired 80% for the prenatal loss endpoint for either the linear (36%) or the logistic (35%) model. Because the single-block design showed such poor performance with respect to prenatal loss, other design options were examined.

#### Single-Block Design, Two F_1_ Females per Litter

In an attempt to increase the power of the experiment without increasing the number of P_0_ dams, a design in which two females per F_1_ litter (*i.e.*, sisters) are bred was examined. A major complication with this unconventional design is that the pups from the litters of the two related F_1_ females would not be independent. The resulting correlation structure is presented in Figure 1b.

Rochon’s [31] sample size methodology is flexible enough to accommodate such a correlation structure. However, this correlation structure requires additional assumptions. First, it must be assumed that the correlation between litters of sisters is the same for all sister pairs. Likewise, it must be assumed that correlation between any pup from one litter and any pup from the related litter is the same for any such pair of pups.

In addition, the size of the inter-litter correlation relative to the intra-litter correlation must be estimated. It is a reasonable assumption that the inter-litter correlation would be smaller than the intra-litter correlation, though it is unclear how much smaller. To address this last issue, a sensitivity analysis could be conducted. The true nature of the correlation structure for such a design is uncertain, and a violation of any one of these assumptions could affect the results. Validation of these assumptions is not possible, because Narotsky *et al.* [27] does not provide data on litters of related females. Moreover, this type of design, breeding more than one F_1_ female per litter, is unconventional and was not encountered in the scientific literature. Despite the likelihood that this design would not be usable for the current study, it was examined to determine its potential usefulness for future studies; if large increases in expected power can be realized with such a design, then pilot data could be collected to provide the required estimates and support validation of the assumptions for future work.

Based on a single value for the relative value of the inter-litter correlation to the intra-litter correlation (δ = 0.75), the results were encouraging. As expected based on the results above with one F_1_ female, power for the pup weight endpoint was greater than 99% with a control:treatment group ratio of either 50:50 or 40:60. Power for the prenatal loss endpoint continued to fall below the desired 80% level, but increased using the linear (43%) and logistic (42%) models, respectively, and with unequal allocation of the dams, as stated above for the one female per litter design.

Despite the increased power using two females per litter, this design was not further considered. This design required more water concentrate than the alternative two-block design (discussed below). In addition, the project team lacked confidence in the method of handling the correlation between related litters and was doubtful that all necessary assumptions would be met. Nonetheless, it is important to note that this approach, *i.e.*, using multiple offspring per litter, can enhance the sensitivity of reproductive toxicity testing for several endpoints (e.g., onset of puberty, estrous cyclicity) [32,33] and warrants further exploration.

### 3.3. Final Design

#### Two-Block Design, with One F_1_ Female per Litter

To achieve the desired level of power without the complication of inter-litter correlation, a design with two blocks of 100 P_0_ dams per block (total of 200 dams) was examined. Although a single-block design lacks the complication of a blocking factor, managing 200 dams in a single block exceeded the technical capability available to conduct the study. A design with two blocks (*i.e.*, replicates) of 100 dams each was logistically more feasible and was expected to produce increases in power dependent upon the size of the group × block interaction effects.

If dams are examined in two blocks, a factor must be included in the model to account for differences between the two blocks. The blocks would be treated as a random factor in the analysis of the data from the multi-generational study being powered here. The model under consideration for pup weight was revised as:

(7) $$y_{ijkl}=\beta_{i}x_{ijk}+\tau_{k}+{\left(\beta \tau \right)}_{ik}\;x_{ijk}+{ɛ}_{ijkl}$$

where *y_ijkl_* is the weight of the *l*^th^ live pup in the *k*^th^ block in the *j*^th^ litter of the *i*^th^ group:

$$x_{ijk}=\{\begin{array}{l} 1\;\text{if the }j^{th}\;\text{observation is from the }k^{th}\;\text{block and the }i^{th}\;\text{group}\\ 0\;\text{otherwise}, \end{array}$$

where *i* equals 1 (control) or 2 (treatment). τ*_k_* is the random effect of the *k*^th^ block, with τ*_k_* ~ *N* (0, *σ_βτ_*^2^), and (*βτ*)*_ik_* is the random effect of the *i*^th^ treatment group by *k*^th^ block interaction, with (&*beta;τ*)*_ik_* ~ *N*(0*, σ_βτ_*^2^). The experiment was designed to include replication so that both the block and the treatment group by block interaction can be estimated.

Using the same notation, the revised model for prenatal loss was:

(8) $$\pi_{ik}=\beta_{i}x_{ijk}+\tau_{k}+{\left(\beta \tau \right)}_{ik}\;x_{ijk}$$

Three different scenarios representing the experimental outcomes were examined in this analysis: (1) the block effect is zero (*i.e.*, no significant block effect), (2) the main effect of block is significant, and (3) the main effect of block and the group × block interaction are significant.

To determine the power achieved for a two-block design, the methodology described by Rochon [31] was used. Implementation of the methodology was similar to that described above for the single-block case, with the same correlation structure used. Parameter estimates required for the algorithm were based on the complex mixture screening-level experiment (Table 1) [27], with the exception of estimates for the block effects, which were not available and, therefore, varied.

To calculate sample size and power for a given scenario with fixed parameters, the algorithm calculations needed to be performed only once. However, because of the random nature of the block effect, a single fixed parameter estimate for the block effect could not be used. Treating the blocking factor as random, the model assumed that the observed block effect was a random observation from the distribution of block effects and that the observed group × block interaction effect was a random observation from the distribution of group × block interaction effects.

To incorporate the random block main effect into the calculations, random noise was generated according to a *N* (0, *σ_τ_*^2^) distribution and added to the group means by block. Random noise was also generated according to a *N* (0, *σ_βτ_*^2^)and added to each group mean to incorporate the random interaction effect. As stated above, the values for *σ_τ_*^2^ and *σ_βτ_*^2^ were varied since no estimates were available. The ranges for these random block effects were selected to provide sufficiently realistic variability without overwhelming the true effects of interest in the model. Power was calculated 500 times and the average power reported.

## 4. Results

### 4.1. Single-Block Design, One F_1_ Female per Litter

**Figure 2:**
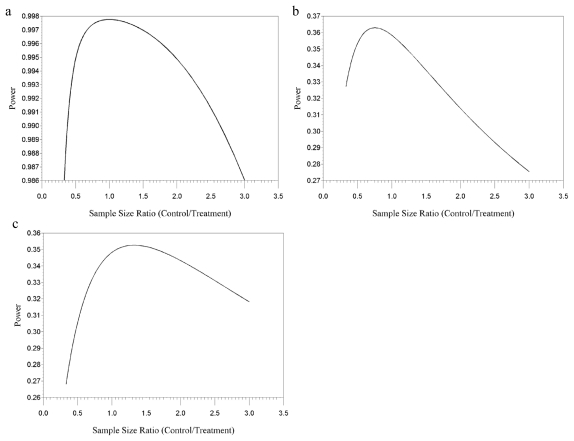
(**a**) Power of the test using single-block design for the pup weight endpoint for varying control-to-treatment sample size ratios assuming combined male and female pups, μ_c_ = 6.5, μ_t_ = 5.9, ρ = 0.60, ψ = 7.59, and *n*_c_ + *n*_t_ = 100; (**b**) Power of the test using single-block design and linear model for the prenatal loss endpoint for varying control-to-treatment sample size ratios assuming μ_c_ = 0.08, μ_t_ = 0.15, ρ = 0.19, ψ = 3.23, and *n*_c_ + *n*_t_ = 100; (**c**) Power of the test using single-block design and logistic model for the prenatal loss endpoint for varying control-to-treatment sample size ratios assuming μ_c_ = 0.08, μ_t_ = 0.15, ρ = 0.19, ψ = 3.23, and *n*_c_ + *n*_t_.

Power as a function of sample size ratio for pup weight is presented in Figure 2a. Results from examination of a single-block design showed that power with respect to pup weight would be maximized with equal allocation of the dams into the two treatment groups; however, it can be seen from Figure 2a that any control/treatment ratio between 0.5 and 2.5 would provide greater than 99% power.

In contrast, for prenatal loss, an unequal allocation of the dams in the single-block design led to increased power by placing more dams in the group anticipated to have greater variance. The recommended allocation differed for the linear and the logistic models for prenatal loss. Power as a function of sample size ratio for the linear and logistic prenatal loss models are presented in Figure 2b and Figure 2c, respectively. For the linear model [with variance, σ^2^, equal to *np* (1 − *p*)], the effect variance for the treatment group, σ^2^ = 1.69, was greater than that for the control group, σ^2^ = 0.96. The reverse was true for the logistic model [with variance, σ^2^, equal to 1/np + 1/n(1-p)], where the effect variance for the treatment group was σ^2^ = 0.59 and for the control group was σ^2^ = 1.04. As a result, power was maximized at a control:treatment group ratio of 43:57 for the linear model and 57:43 for the logistic model. The power difference between the two models was small: at the 1:1 ratio, power was 36% and 35% for the linear and logistic models, respectively.

### 4.2. Two-Block Design, One F_1_ Female per Litter

For pup weight in the two-block design with equal allocation to the control and treatment groups, the use of 100 dams in each of the two blocks was sufficient to achieve the desired 80% power with a significance level of 0.05. For this optimal allocation, mean power (calculated over 500 values for the block effect and the group × block effect) was approximately 100% to detect the treatment effect in 54% of the scenarios, greater than 90% for 62% of the scenarios, greater than 85% for 92% of the scenarios, and greater than 80% for 100% of the scenarios. Moreover, median power to detect pup weight differences was approximately 100% for all scenarios examined. These results held true for the different numbers of live pups per litter, as well as for each sex separately (results not shown) and for both sexes combined.

Based on the results from the single-block designs, two control:treatment ratios representing unequal allocation were considered for the two-block design, along with an equal allocation ratio: 45:55, 40:60, and 50:50. Pup weight results for three potential experimental outcome scenarios are represented in Table 2. Row 1, with block and interaction variance both equal 0, represents the no significant block effect case. Rows 2–4 represent the significant main block effect (block effect variance > 0) but no group x block interaction (interaction effect variance = 0). The remaining rows represent cases where both the main effect of block and the block x group interaction are significant since both variances are >0. Table 2 presents the power results for the pup weight endpoint for the combined male pups and female pups for the two-block design with 40:60 ratio. Both mean and median power are presented in Table 2 as the distribution was skewed. These results are equivalent to the results for the 45:55 and 50:50 ratios. For more than half of the scenarios considered (54%), the mean power is 100% to detect the treatment effect with respect to the pup weight endpoint; 62% of the cases achieve at least 90% mean power, 92% of cases achieve at least 85% mean power, and all cases (100%) achieve at least 80% mean power. Median power was approximately 100% to detect pup weight differences for all scenarios examined.

For prenatal loss, two blocks of 100 dams with equal allocation to the control and treatment groups appeared to be insufficient to achieve the desired 80% power at a significance level of 0.05. The two-block design with a 40:60 ratio (control:treatment) of the dams within each block yielded the highest power estimates for prenatal loss; however, power remained below the desired 80%. The power results for prenatal loss are given in Table 3 for the linear model and Table 4 for the logistic model; power to detect a statistically significant treatment effect with respect to prenatal loss never exceeded 60%. The linear model yielded slightly better mean power estimates of 57–58% to detect 7.1 percentage point differences in prenatal loss for 13 implantation sites per dam.

In Tables 3 and 4, which are structurally similar to Table 2 as described above for prenatal loss, power tended to be lower assuming 16 implants per dam compared to 13 implants per dam. Recall the over-dispersion factor is a function of the number of implants per dam assumed, and over-dispersion increases as this value increases. Increased over-dispersion corresponds to increased variance, which results in decreased power.

## 5. Discussion

This article describes the methodology used for calculating statistical power for non-independent observations in a two-block design for the multigenerational reproductive/developmental toxicity rodent bioassay in the Four Lab Study. It takes into account the multigenerational bioassay design as well as constraints on sample size, water concentrate volume and concentration factor.

Designing this bioassay under these constraints necessitated thoughtful consideration of statistical power. Determining power and sample size for multiple block designs is complicated, because it must account for the interaction of groups with blocks. Though the effect of the block is not of inherent interest, it might influence the group (treatment) effect if the DBP levels within the concentrate change during the course of the study. Using developmental toxicity screening data [27], power calculations were made for prenatal loss and pup weight. These endpoints were considered of primary importance because they correspond to effects reported in positive epidemiologic studies [9,10,13,14].

While several rodent developmental toxicity investigations have been conducted using exposures to concentrated tap waters [15,27,34,35], only Uriu-Hare *et al.* [35] reported power calculations. They calculated the sample size necessary to provide 80% power to detect a 50% increase in the number of dams with at least one resorption. The authors subsequently found a significant increase in the incidence of dams with one or more resorptions among those dams drinking unconcentrated tap water compared to dams drinking deionized water. The power calculations developed for this multigenerational bioassay are more detailed than those of Uriu-Hare *et al.* [35]. Other DBP toxicology studies to date show a lack of adverse developmental effects or only marginal or subtle effects on rodent development, however, without estimates of power, these results are difficult to interpret [15,24,34,35].

Based on the results of the power analyses and the physical constraints of the study, the Four Lab team selected a two-block design for the multigenerational bioassay, assigning 40 and 60 timed-pregnant rats to the control and treatment groups in each block, respectively. The two-block design achieved greater than the desired 80% power at a significance level of 0.05 with respect to pup weight for all the scenarios examined; more than half of the scenarios for the two-block design achieved 100% power. This design ensured that at least one sensitive endpoint (*i.e.*, pup weight) had maximum power to detect an effect at the low-response region of the dose-response curve. Fetal or pup weight is known to be a sensitive developmental endpoint in animal studies that is often observed at doses below those causing other developmental effects [36]. This endpoint is also relevant to human health, as low birth weight children have been shown to be at increased risk for developing several chronic sequelae [37,38].

For this research, detecting a prenatal loss effect, if present, also was desirable. The two-block design, that optimizes the power for pup weight loss, also provides the most power for prenatal loss from among the possible designs, given the study constraints. This analysis shows that the two-block design provides a modest amount of power (*i.e.*, at most, 57–58% using the linear model) to detect differences in prenatal loss. Analyses showed that the simplest design, consisting of a single block, would have insufficient power. A second possible single-block design, which bred two females per F_1_ litter, was eliminated from consideration due to the uncertainty surrounding the inter-litter correlation, and the need for larger quantities of water concentrate. In general, the power associated with the two-block design was approximately twice that of the single-block designs considered. The somewhat large discrepancy between the power for pup weight and prenatal loss was expected and is inevitable in a reproductive toxicity study. This is because, relative to their respective means, the variance for prenatal loss is generally much larger than for pup weight (e.g., Table 1). Thus, based on this analysis, the two-block design was considered the best design option for the Four Lab multigenerational bioassay.

The constraints imposed by conducting toxicological investigations with highly complex environmental mixtures in an environmentally relevant medium at environmentally relevant dose levels are not unique to the Four Lab Study. The methodology described here may be applied to appropriately design other toxicology studies with environmentally realistic complex mixtures, as similar constraints likely will be encountered.

This work highlights the importance of considering statistical power in the design of bioassays that evaluate health effects of chemical mixtures in the low-response region of the dose-response curve. Such biostatistical analyses provide meaningful quantitative insights into the trade-offs inherent in the design of studies conducted in the low-response region and provide a clear and logical rationale for choice of study design. These analyses and insights lead to toxicological studies in the low-response region that provide meaningful results and allow for appropriate interpretation of experiments when no observable adverse effect is detected. The conduct of such toxicological studies is critical for improved dose-response assessments of complex chemical mixtures, because they increase understanding of the potential human health effects from exposure to chemical mixtures near environmental exposure levels, which are of increased relevance to human health risk assessment [39].

## Figures and Tables

**Table 1:** Summary of data from pilot study with complex mixture of disinfection by-products [27].

	Control	Treated
**No. dams**	36	35
**No. live litters**	36	35

	**Mean ± S.E. per litter**	

**No. implantation sites**	13.1 ± 0.3	13.4 ± 0.4
**No. live pups**	12.1 ± 0.4	11.3 ± 0.6
**Prenatal loss (%)**	7.8 ± 1.5	14.9 ± 3.8
**Pup weight (g)**	6.5 ± 0.1	5.9 ± 0.1^a^

a Significantly different from controls (*p* < 0.01).

**Table 2:** Power to detect a 0.6 g difference in average pup weight using a linear model: Two-block design.

		Mean Power		Median Power	

Block effect variance, *σ*^2^_τ_	Interaction effect variance, *σ*^2^*_β_*_τ_	12 live pups/litter	15 live pups/litter	12 live pups/litter	15 live pups/litter
0.00	0.00	1.00	1.00	1.00	1.00
0.05	0.00	1.00	1.00	1.00	1.00
0.5	0.00	1.00	1.00	1.00	1.00
1.0	0.00	1.00	1.00	1.00	1.00
0.05	0.05	1.00	1.00	1.00	1.00
0.05	0.5	0.84	0.84	1.00	1.00
0.05	1.0	0.86	0.86	1.00	1.00
0.5	0.05	1.00	1.00	1.00	1.00
0.5	0.5	0.86	0.86	1.00	1.00
0.5	1.0	0.91	0.91	1.00	1.00
1.0	0.05	1.00	1.00	1.00	1.00
1.0	0.5	0.85	0.86	1.00	1.00
1.0	1.0	0.89	0.89	1.00	1.00

Note: Calculated across 500 simulations, assuming one F1 female per dam is bred, combined male and female pups, and unequal allocation of dams to control (40) and treatment (60) groups within each of the two blocks. Assumes an individual two-sided test, significance level of 0.05. Control group average pup weight = 6.5. Treatment group average pup weight = 5.9. ρ = 0.60. ψ = 7.59 or 9.39.

**Table 3:** Power to detect a 7.1 percentage point difference in prenatal loss using a linear model: Two-block design.

		Mean Power		Median Power	

Block effect variance, *σ*^2^_τ_	Interaction effect variance, *σ*^2^_βτ_	13 implants/dam	16 implants/dam	13 implants/dam	16 implants/dam
0.00	0.00	0.57	0.53	0.57	0.53
0.001	0.00	0.57	0.53	0.57	0.53
0.01	0.00	0.57	0.53	0.57	0.53
0.025	0.00	0.58	0.54	0.57	0.53
0.001	0.001	0.57	0.53	0.57	0.53
0.001	0.01	0.57	0.53	0.57	0.53
0.001	0.025	0.57	0.54	0.58	0.54
0.01	0.001	0.57	0.53	0.57	0.53
0.01	0.01	0.57	0.54	0.57	0.53
0.01	0.025	0.57	0.54	0.57	0.53
0.025	0.001	0.58	0.54	0.57	0.53
0.025	0.01	0.58	0.54	0.57	0.53
0.025	0.025	0.57	0.54	0.57	0.53

Note: Calculated across 500 simulations, assuming one F1 female per dam is bred, a linear model, and unequal allocation of dams to control (40) and treatment (60) groups within each of two blocks. Assumes an individual one-sided test, significance level of 0.05. Control group prenatal loss = 0.08. Treatment group prenatal loss = 0.15. ρ = 0.19. ψ = 3.23 or 3.79.

**Table 4:** Power to detect a 1.9-fold difference in prenatal loss using a linear logistic model: Two-block design.

		Mean Power		Median Power	

Block effect variance, *σ*^2^_τ_	Interaction effect variance, *σ*^2^_βτ_	13 implants/dam	16 implants/dam	13 implants/dam	16 implants/dam
0.00	0.00	0.52	0.48	0.52	0.48
0.001	0.00	0.52	0.48	0.52	0.48
0.01	0.00	0.52	0.48	0.52	0.48
0.025	0.00	0.52	0.48	0.52	0.48
0.001	0.001	0.52	0.48	0.52	0.48
0.001	0.01	0.53	0.49	0.53	0.49
0.001	0.025	0.54	0.50	0.54	0.51
0.01	0.001	0.52	0.48	0.52	0.48
0.01	0.01	0.51	0.48	0.51	0.48
0.01	0.025	0.51	0.47	0.51	0.49
0.025	0.001	0.52	0.48	0.52	0.48
0.025	0.01	0.52	0.49	0.52	0.48
0.025	0.025	0.51	0.48	0.51	0.48

Note: Calculated across 500 simulations, assuming one F1 female per dam is bred, a logistic model, and unequal allocation of dams to control (40) and treatment (60) groups within each of two blocks. Assumes an individual one-sided test, significance level of 0.05. Control group prenatal loss = 0.08. Treatment group prenatal loss = 0.15. ρ = 0.19. ψ = 3.23 or 3.79.
